# Caring Contacts to Reduce Psychiatric Morbidity Following Hospitalization During the COVID-19 Pandemic: A Pilot Randomized Controlled Trial

**DOI:** 10.1177/07067437221121111

**Published:** 2022-08-22

**Authors:** Sarah Holman, Rosalie Steinberg, Mark Sinyor, Hillary Lane, Kaleigh Starritt, Sidney H. Kennedy, Thomas Niederkrotenthaler, Ari Zaretsky, Saulo Castel, Ayal Schaffer

**Affiliations:** 1 Department of Psychiatry, 71545 Sunnybrook Health Sciences Centre, Toronto, Ontario, Canada; 2 Department of Psychiatry, 7938 University of Toronto, Toronto, Ontario, Canada; 3 Department of Psychiatry Patient and Family Advisory Council, Sunnybrook Health Sciences Centre, Toronto, Ontario, Canada; 4 10071 Department of Psychiatry, St. Michael's Hospital, Toronto, Ontario, Canada; 5 Department of Social and Preventive Medicine, Medical University of Vienna, Vienna, Austria

**Keywords:** caring contact, suicide prevention, psychiatric hospitalization, COVID-19 pandemic

## Abstract

**Objectives:**

Caring Contacts are an emerging intervention that aims to reduce distress and suicide risk after acute psychiatric care. This trial aimed to determine whether, during a pandemic, there was any evidence that the mental health benefits and reduction in suicidal ideation (SI) associated with delivering Caring Contacts to recently discharged psychiatric patients were greater than a control communication. The secondary objective was to identify whether the predicted benefits were greater among people living alone or those diagnosed with depression.

**Method:**

A single-site pilot randomized clinical trial (*n* = 100), with patients recruited from the adult Inpatient Psychiatry Unit at Sunnybrook Health Sciences Centre, Toronto, Canada between August 2020 and May 2021. Participants were randomized (1:1) to the Caring Contact or control group. Participants received three Caring Contact or control communications via email or mail (on days 4, 21, and 56 post-discharge). Mental health symptoms were assessed using the self-report Hopkins Symptom Checklist-25 (HSCL-25) scores at discharge (baseline) and when participants received each communication. Analysis of variance was used for the primary comparisons and exploratory analyses for subgroups.

**Results:**

Both groups experienced a significant worsening of mental health symptoms at all time points post-discharge relative to baseline. There were no significant differences between groups at any time point, however, on day 4 there was a 24.2% and 72.6% attenuated worsening in the Caring Contact group compared to the control group for total symptom severity and SI, respectively. There was no significant interaction effect for the depression subgroup or those living alone.

**Conclusions:**

While this pilot study was not powered to identify significant differences between groups, results are indicative of feasibility and acceptability of the intervention and provide some indication that Caring Contacts may have benefited patients in the days following discharge, supporting the need for larger-scale trials. The study was registered with clinicaltrials.gov (study ID NCT04456062).

## Abrégé

**Objectifs:**

Les contacts attentionnés sont une nouvelle intervention qui vise à réduire le risque de détresse et de suicide après des soins intensifs psychiatriques. Le présent essai visait à déterminer si, durant une pandémie, des données probantes indiquaient que les avantages pour la santé mentale et la réduction de l’idéation suicidaire (IS) associés à la prestation de soins attentionnés à des patients psychiatriques récemment mis en congé étaient plus puissants que le contrôle des communications. L’objectif secondaire était d’identifier si les avantages escomptés étaient plus grands chez les personnes vivant seules ou chez celles où la dépression était diagnostiquée.

**Méthode:**

Un essai clinique randomisé (n = 100) d’un seul site, dont les patients étaient recrutés à l’Unité de psychiatrie pour patients hospitalisés adultes du Centre des sciences de la santé Sunnybrook de Toronto, Canada, entre août 2020 et mai 2021. Les participants ont été randomisés (1:1) aux Contacts attentionnés ou au groupe témoin. Les participants ont reçu trois communications des Contacts attentionnés ou des témoins par courriel ou courrier (aux jours 4, 21, 56 après-congé). Les symptômes de santé mentale ont été évalués à l’aide des scores auto-déclarés à la liste de contrôle des symptômes de Hopkins-25 (HSCL-25) au congé (à la base) et quand les participants ont reçu chaque communication. ANOVA a été utilisée pour les comparaisons primaires, et les analyses exploratoires pour les sous-groupes.

**Résultats:**

Les deux groupes ont connu une aggravation significative des symptômes de santé mentale à différents moments après le congé relativement à la base. Il n’y avait pas de différences significatives entre les groupes à tout moment, cependant au jour 4 il y avait une aggravation atténuée de 24,2% et de 72,6% dans le groupe des Contacts attentionnés comparé au groupe témoin pour la gravité totale des symptômes et les IS respectivement. Il n’y avait pas d’effet d’interaction significatif pour le sous-groupe de la dépression ou de ceux vivant seuls.

**Conclusions:**

Bien que cette étude pilote n’ait pas été mandatée pour identifier les différences significatives entre les groupes, les résultats indiquent la faisabilité et l’acceptabilité de l’intervention et offrent certaines indications à l’effet que Contacts attentionnés puissent avoir été bénéfiques pour les patients dans les jours suivant le congé, appuyant ainsi le besoin d’essais à plus grande échelle. L’étude a été enregistrée à clinicaltrials.gov (ID de l’étude NCT04456062).

## Introduction

The time after discharge from psychiatric hospitalization is a period of increased risk for suicide.^1,2^ Currently, there are few systematic interventions to mitigate this risk and provide support following discharge from psychiatric inpatient care.^3^ Caring Contacts have evolved as an evidence-based solution since 1976.^4,5^ Caring Contacts are brief communications sent to patients post-discharge that convey messages of hope, support, and provide resource information.^4,6^ Studies report that Caring Contacts can reduce suicidal ideation (SI) and behaviour,^6,7^ but to our knowledge, when this study was conducted, Caring Contacts had yet to be evaluated in Canada.

The potential gap in mental health care during the post-discharge period may be amplified for individuals during a pandemic, a time of mass self-isolation when usual supports are impacted.^8–10^ While at the beginning of the COVID-19 pandemic the number of psychiatric admissions decreased in Canada,^11^ individuals with pre-existing psychiatric conditions were especially vulnerable to worsening mental health.^12^

The primary objective of our pilot study was to determine whether, during a pandemic, there was evidence of a greater reduction in depressive and anxiety symptoms associated with delivering Caring Contacts to recently discharged psychiatric inpatients compared to control communications. Planned exploratory objectives were to determine whether the predicted benefits of Caring Contacts were larger during periods of mandatory self-isolation, among people living alone, and those diagnosed with depression. An additional exploratory objective was to also examine changes in SI. We hypothesized that there would be a greater reduction in depressive and anxiety symptoms among patients receiving Caring Contacts compared to those receiving a control communication. For our exploratory objectives, we hypothesized that there would be greater benefits for participants during mandatory self-isolation periods, participants living alone, and participants with a diagnosis of depression.

## Methods

### Design

The study design was a single-site parallel-group randomized clinical trial examining for differences associated with receiving a series of Caring Contact communications compared to control communications among patients recently discharged from a psychiatry inpatient unit. Informed written consent was obtained from all participants. The Research Ethics Board of Sunnybrook Health Sciences Centre approved the study. The study was registered with clinicaltrials.gov (study ID NCT04456062).

### Setting

All participants were recruited from the adult Inpatient Psychiatry Unit at Sunnybrook Health Sciences Centre, a large university-affiliated hospital in Toronto, Canada. Recruitment started on August 4, 2020, and was completed on May 5, 2021. The last participant received the final correspondence on July 16, 2021.

### Participants

Participants were recruited based on the following inclusion criteria: (1) aged 18 or above; (2) inpatient status at the time of recruitment; (3) having an email or mailing address; (4) ability to read and understand English; and (5) capacity to provide informed consent and ability to understand and comply with the requirements of the study. The only exclusion criterion was a diagnosis of a major neurocognitive disorder. Eligible patients were identified using the hospital's Patient Care System (Quadramed) or were referred from the inpatient service and given the opportunity to participate. The target sample size had to be reduced from 150 to 100 participants because of inherent challenges in conducting a clinical study within a hospital setting during the COVID pandemic (e.g., closures due to outbreaks and restrictions on research staff allowed in the hospital). This shifted the trial from being fully powered to a pilot study.

### Intervention

The content, delivery method, and timing of the Caring Contact messages were informed by preliminary work with patients and staff on the inpatient unit. The Caring Contact communication consisted of a positive message of hope and support with words of encouragement, a reminder of clinical and emergency resources, and a link to the Hopkins Symptom Checklist-25 (HSCL-25) symptom questionnaire. The control communication included the same link to the symptom questionnaire (HSCL-25) and emergency resources, but no positive message. Since the only difference was in the nature of the content (i.e., the presence of a positive message), some degree of subject blinding could be maintained. However, this was not formally tested as it was not considered true blinding. Participants received a total of three Caring Contact/control communications, the first on day 4 post-discharge, the second on day 21, and the third on day 56. Each Caring Contact (day 4, day 21, and day 56) had a different positive message which remained consistent across all participants in the Caring Contact group. The control communication was the same at all time points. See supplementary materials for Caring Contact and control communications. Prior to discharge, all participants received an emergency contacts information sheet.

Participants were given the option to receive the Caring Contact/control communication via email or by regular mail. At enrollment, 93% of participants opted to receive communications by email. Four participants later switched from email to mail, and 1 switched from mail to email. If participants chose the mailing option, the symptom questionnaire (HSCL-25) and a pre-paid return envelope was included for return back to the research team.

### Procedures

Eligible inpatients were approached by research staff. After participants consented, they were block randomized with a random allocation sequence generated by an allocation table through the Research Electronic Data Capture (REDCap) study platform in a 1:1 ratio to the Caring Contact intervention or control group. Research staff were unaware of the allocation sequence. Participants were never informed which group they were in, however, they were aware of the difference between the groups and may have noticed once they received the communications. Study data were collected and managed using REDCap tools hosted at the Sunnybrook Research Institute.^13,14^ Participants completed a demographic questionnaire at enrollment and the HSCL-25 within 4 days prior to discharge, which served as a baseline measure. Research staff obtained participant medical information from Quadramed to complete a health history questionnaire.

**Figure 1.**
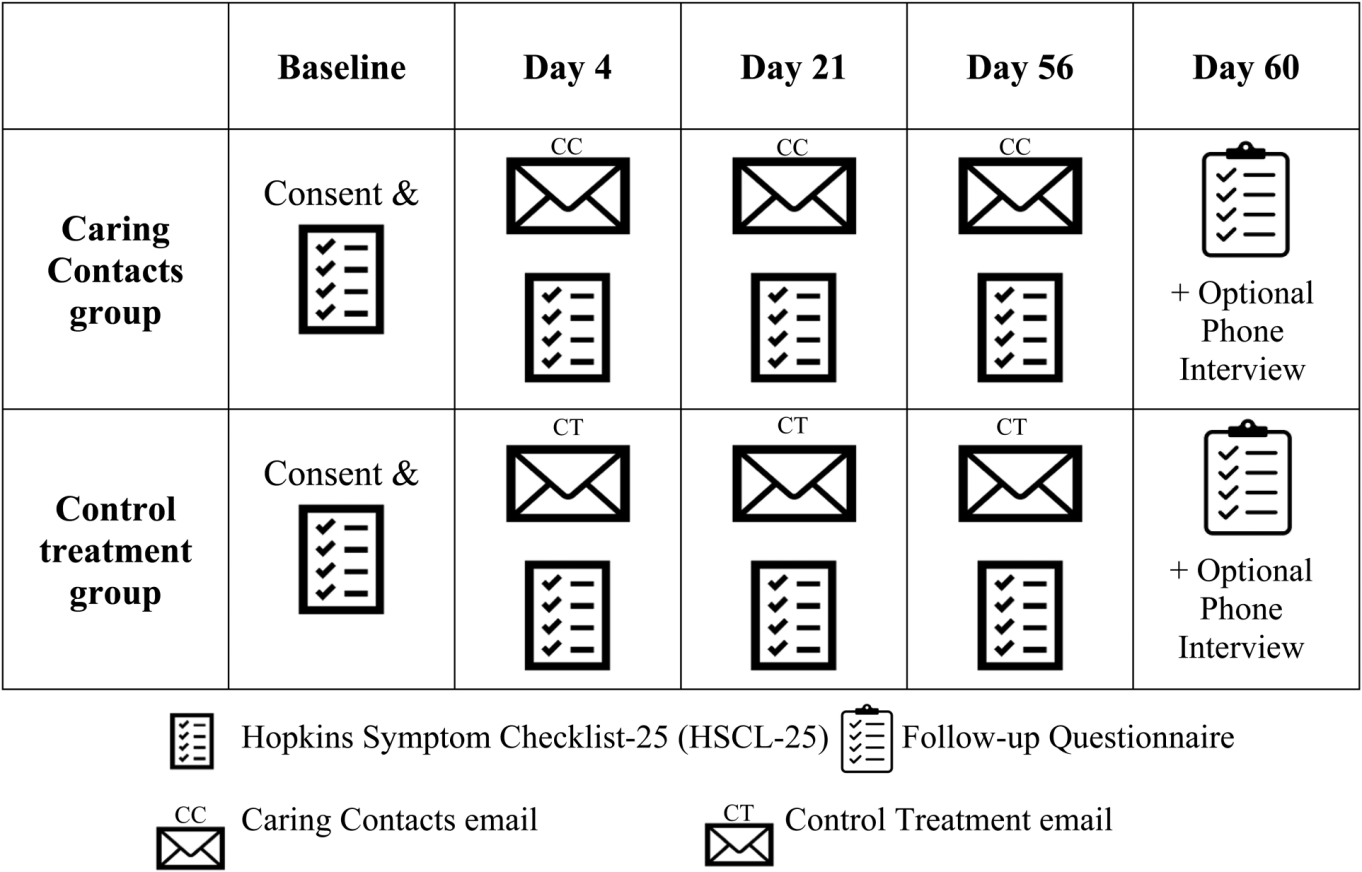
Study Outline.

The study outline is displayed in Figure 1. Participants who did not complete the HSCL-25 within 4 days of receiving each communication were sent an automatic reminder email. If participants did not respond to the day 4 questionnaire within 14 days post-discharge, they received a reminder phone call from research staff.

There was an optional follow-up questionnaire sent on day 60 post-discharge for participants to provide feedback about their experience in the study. In this questionnaire, participants could indicate if they wanted to be contacted for a 20-minute follow-up interview via phone to provide more detailed feedback. Participants who agreed were contacted and interviewed by research staff within 3 weeks.

### Measures

#### Primary Outcome Measure

The primary outcome measure was the total score on the HSCL-25, which is a widely-used self-report Likert scale-based questionnaire measuring symptoms of anxiety and depression.^15^ There are 25 items on the questionnaire and the scale ranges from 1 = “Not at all,” 2 = “A little,” 3 = “Quite a bit,” and 4 = “Extremely.” The scores for each item were summed for a total score with a minimum of 25 and a maximum of 100. Scores from each time point (day 4, day 21, and day 56) were used to assess the efficacy of the intervention. Group means were calculated to conduct analyses. One of the items on the HSCL-25 included “thoughts of ending your life.” Ratings of this item were used in an exploratory manner to assess for changes and differences in SI within and between groups.

#### Supplementary Measures

The demographic questionnaire included age, gender, marital status, racial or ethnic group, and sexual orientation. The health history questionnaire included discharge date, length of hospital stay, whether this was the first-lifetime psychiatric admission, number of psychiatric admissions in the past year, number of lifetime psychiatric admissions, primary psychiatric diagnosis, comorbid psychiatric diagnoses, suicide risk identified as a reason for admission, suicide attempt in the context of admission, suicide attempt in the past year, lifetime suicide attempt, SI at time of discharge, scheduled to see a psychiatrist/family physician/therapist or other psychosocial supports/peer support post-discharge, and psychiatric follow-up at Sunnybrook.

The follow-up questionnaire and interview included questions regarding the content and delivery of the communications. While these results are not reported in this article, this feedback was collected to refine the intervention for broad clinical use.

### Statistical Analysis

We used the last observation carried forward (LOCF) method for missing data points on the HSCL-25. To test our primary hypothesis, a mixed-model analysis of variance (ANOVA) was conducted on the HSCL-25 scores with time point (baseline, day 4, day 21, and day 56) as the within-subjects factor and group (Caring Contact vs. Control) as the between-subjects factor. Bonferroni adjustment for multiple comparisons was applied to the ANOVA. Visual inspection of box plots, histograms, and Q–Q plots was completed. The ANOVA and assumptions of normality, homogeneity of variance, and sphericity were all assessed. To investigate our exploratory objectives we included additional between-subjects factors for each subgroup of interest (e.g., participants with depression) in separate mixed-model ANOVAs. We used a nonparametric test to examine changes over time in the SI item on the HSCL-25 for each group. To examine between-group differences in demographic and psychiatric history variables, we conducted *t*-tests and chi-squares. IBM SPSS Statistics for Windows, version 28, was used for all analyses.

## Results

### Participants

**Figure 2.**
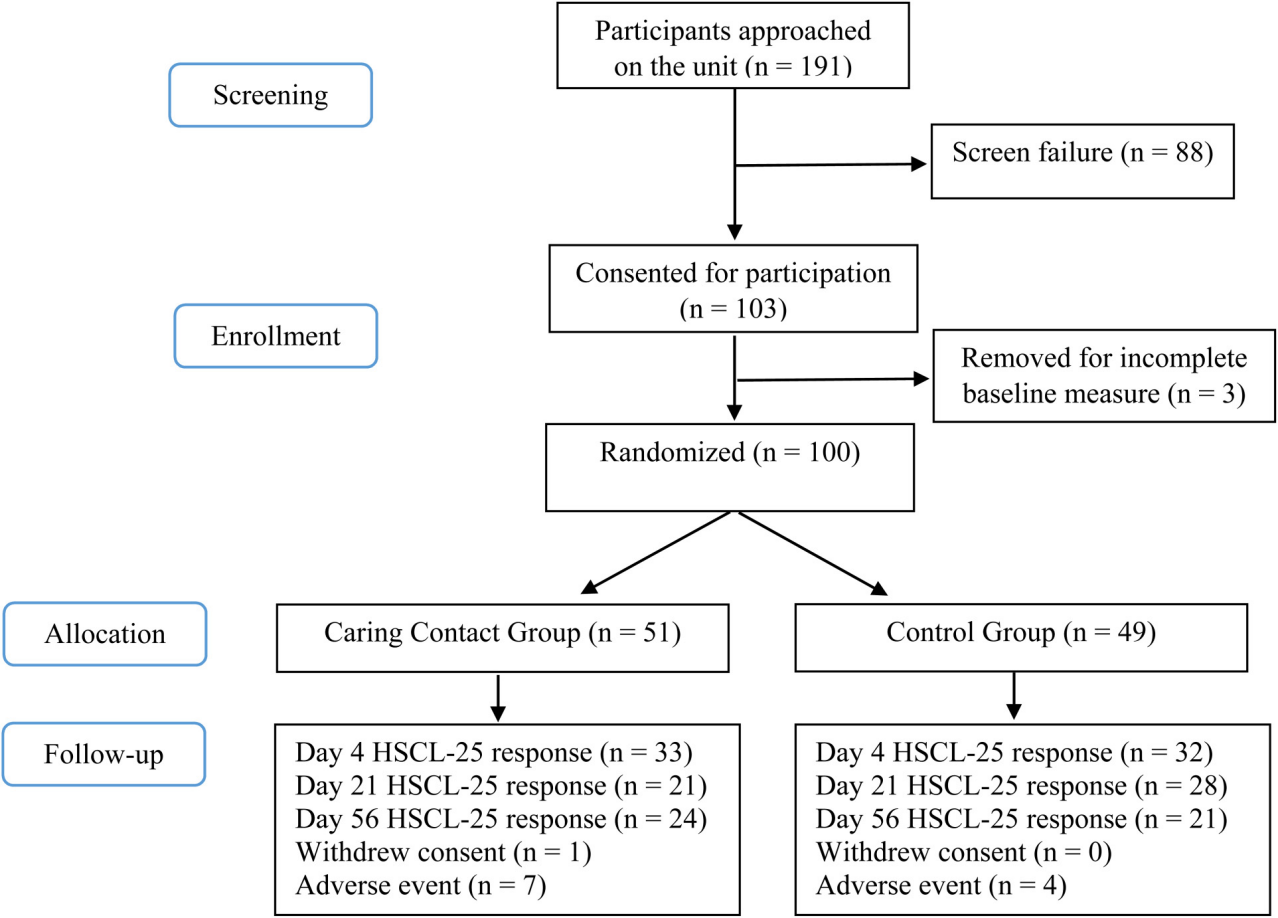
CONSORT Flow Chart.

A total of 100 participants were randomized in the study. The screening and study enrollment are illustrated in the CONSORT flowchart (Figure 2). A total of 12 participants were withdrawn from the study, 11 due to re-hospitalization at Sunnybrook and 1 at the participant's request. Prior to their withdrawal, 6 participants received 1 communication and 6 participants received 2 communications.

The participants’ age range was 18-77 years. Demographics for each group are included in Table 1. The Caring Contact group had significantly more participants who were divorced/separated/widowed while the control group had more participants who were married/living common law. Other than marital status, there were no statistically significant group differences. The most common primary psychiatric diagnosis in our sample was a depressive illness (29%), followed by bipolar disorder (28%), psychotic disorders (24%), substance use disorders (7%), personality disorders (6%), anxiety disorders (3%), and stressor-related disorders (3%). See Table 2 for participants’ psychiatric history.

**Table 1.** Patient Demographics.

	Caring Contact (*n*** **=** **51)	Control (*n*** **=** **49)	Between-group statistical test for differences
Age (mean, years)	36.6	38.7	*P* = 0.44
Gender			*P* = 0.12
Female	22	28	
Male	26	21	
Other	3	—	
Race			*P* = 0.78
White	25	23	
Asian	10	7	
Black	3	5	
Other	13	14	
Marital status			*P* < 0.01
Single (never married)	28	27	
Married/living common law	7	18	
Divorced/separated/widowed	12	3	
Chose not to disclose	4	1	
Sexual orientation			*P* = 0.15
Heterosexual	35	38	
Bisexual	8	2	
Gay/Lesbian/Queer	2	5	
Chose not to disclose	6	4	

**Table 2.** Participant Psychiatric History.

	Caring Contact	Control	Between-group statistical test for differences
Length of hospital stay (mean)	17.2 days	23.0 days	*P* = 0.69
Number of participants with current admission as the first admission	25	23	*P* = 0.59
Total admissions past year (mean)	2	1.9	*P* = 0.68
Total admissions life (mean)	4.7	6.0	*P* = 0.36
Suicide risk as a reason for admission			*P* = 0.54
Yes	20	23	
No	31	26	
Suicide attempt in the context of admission			*P* = 0.79
Yes	9	7	
No	42	42	
Suicide attempts in the past year			*P* = 0.56
Yes	10	10	
No	40	36	
Unknown	1	3	
Suicide attempt lifetime			*P* = 0.97
Yes	19	17	
No	31	31	
Unknown	1	1	
Suicidal ideation at the time of discharge			*P* = 0.37
Yes	0	1	
No	50	48	
Unknown	1	-	
Seeing a psychiatrist post-discharge			*P* = 0.25
Not at all	4	2	
Within 7 days	23	31	
Within 30 days	23	16	
Unknown	1	-	
Seeing a family physician post-discharge			*P* = 0.47
Not at all	33	37	
7 days	3	1	
30 days	14	11	
Unknown	1	-	
Seeing a therapist or other psychosocial supports post-discharge			*P* = 0.53
Not at all	20	25	
7 days	1	1	
30 days	29	23	
Unknown	1	—	
Peer support post-discharge			*P* = 0.36
Yes	1	3	
No	49	46	
Unknown	1	—	
Psychiatric follow-up at Sunnybrook			*P* = 0.83
Yes	35	35	
No	16	14	

### Primary Outcomes

**Figure 3.**
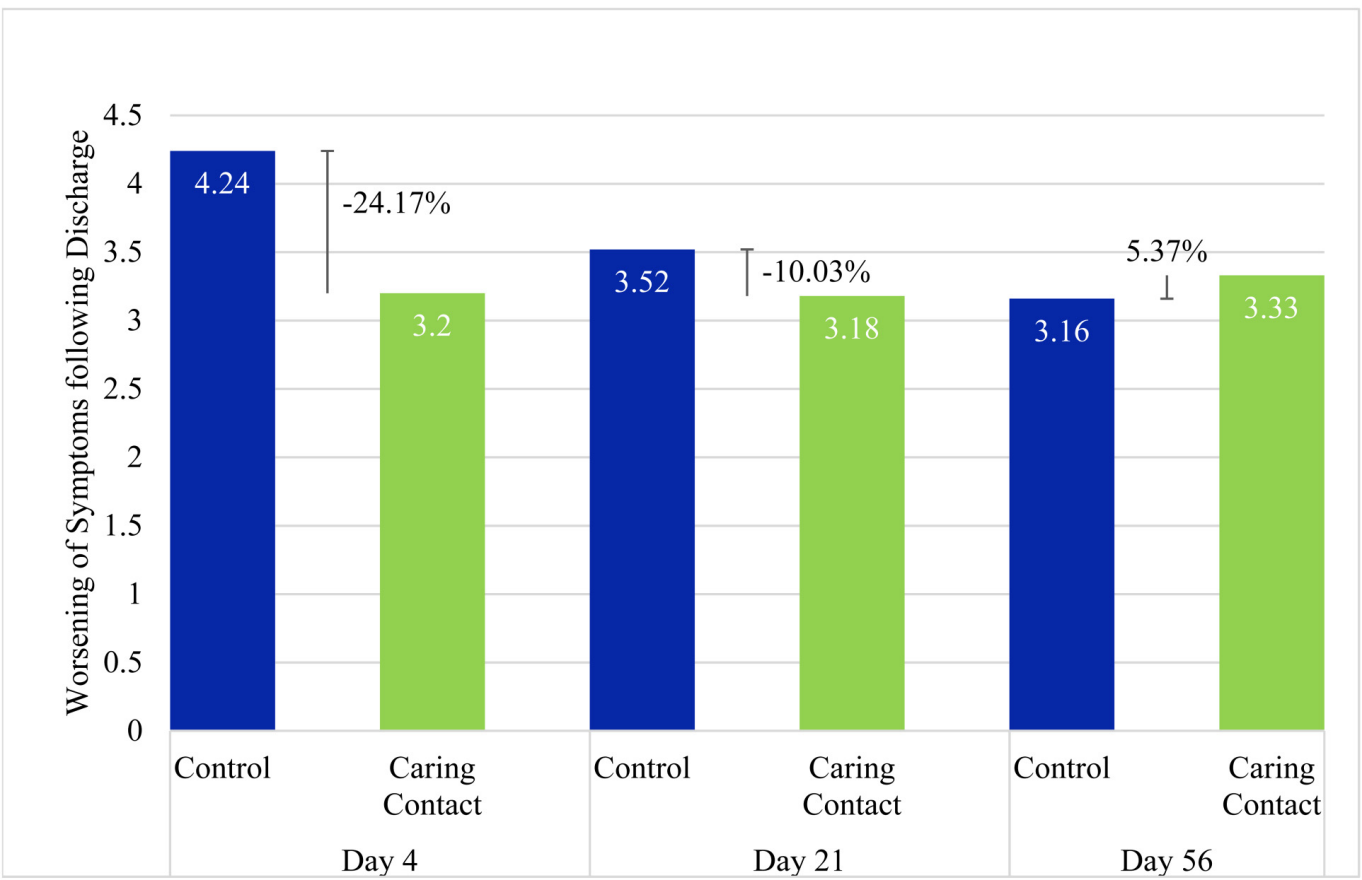
Increase of Total HSCL-25 Score from Baseline.

Mean total HSCL-25 scores for the Caring Contact group increased from a baseline score of 45.3 (*SD* = 15.6) to 48.5 (*SD* = 16.3) on day 4, 48.5 (*SD* = 16.4) on day 21, and 48.6 (*SD* = 16.5) on day 56. The control group also increased from a baseline score of 44 (*SD* = 15.8) to 48.2 (*SD* = 16.7) on day 4, 47.5 (*SD* = 16.4) on day 21, and 47.1 (*SD* = 18.3) on day 56. The mixed-model ANOVA for the HSCL-25 total score data revealed a significant main effect of time (*F*_2.2,217.6_ = 7.8, *P* < 0.01). Post hoc comparisons revealed a significant difference between baseline and day 4 (MD = −3.72 [95% CI −6.19 to −1.26], *P* < 0.01), baseline and day 21 (MD = −3.35 [95% CI −6.09 to −0.62], *P* = 0.01), baseline and day 56 (MD = −3.25 [95% CI −6.25 to −0.25], *P* = 0.03). There were no significant differences between day 4 and day 21 (MD = 0.37 [95% CI −1.29 to 2.03], *P* = 1.0), day 4 and day 56 (MD = 0.47 [95% CI −1.77 to 2.71], *P* = 1.0), or day 21 and day 56 (MD = 0.11 [95% CI −1.67 to 1.88], *P* = 1.0). There was no significant main effect of group (*F*_1,98_ = 0.11, *P* = 0.74, MD = 1.03 [95% CI −5.17 to 7.22]) and no significant interaction effect between group and time (*F*_2.2,217.6_ = 0.19, *P* = 0.85). See Figure 3 for group change scores and percent change of the Caring Contact group relative to the control group.

Percentages shown represent the percent change relative to the control group.

There were three high-score outliers in the Caring Contact group at baseline. Removing these outliers did not change the ANOVA results therefore we opted to include the outliers in the dataset. Mauchly's test of sphericity was significant and the Greenhouse–Geisser correction was applied. Kolmogorov–Smirnov tests of normality were significant. Given the sensitivity of these tests and the robust nature of the ANOVA to slight deviations in normality, we also assessed normality by visual inspection of box plots, histograms, and Q–Q plots and determined the data was not badly skewed.

### Secondary Outcomes

The mixed-model ANOVA with an additional between-subjects factor of a primary or secondary diagnosis of depression (*n* = 36) revealed a significant main effect of time (*F*_2.2,213.5_ = 7.24, *P* < 0.01). Post hoc comparisons revealed a significant difference between baseline and day 4 (MD = −3.69 [95% CI −6.28 to −1.09], *P* < 0.01), baseline and day 21 (MD = −3.40 [95% CI −6.29 to −0.52], *P* = 0.01), baseline and day 56 (MD = −3.42 [95% CI −6.58 to −0.26], *P* = 0.02). There were no significant differences between day 4 and day 21 (MD = 0.28 [95% CI −1.47 to 2.03], *P* = 1.0), day 4 and day 56 (MD = 0.26 [95% CI −2.09 to 2.61], *P* = 1.0), or day 21 and day 56 (MD = −0.02 [95% CI −1.87 to 1.84], *P* = 1.0). There was no significant main effect on the treatment group (*F*_1,96_ = 0.0, *P* = 0.97, MD = 0.0 [95% CI −6.34 to 6.57]) or the depression group (*F*_1,96_ = 79, *P* = 0.38, MD = −2.88 [95% CI −9.34 to 3.58]). There was no significant interaction effect between the treatment group and depression group (*F*_1,96_ = 1.37, *P* = 0.25), treatment group and time (*F*_2.2,213.5_ = 0.22, *P* = 0.83), depression group and time (*F*_2.2,213.5_ = 0.27, *P* = 0.80), and between time, treatment group, and depression group (*F*_2.2, 213.5_ = 0.06, *P* = 0.95).

The mixed-model ANOVA with an additional between-subjects factor of living alone (*n* = 23) revealed a significant main effect of time (*F*_2.2, 213_ = 4.60, *P* = 0.01). Post hoc comparisons revealed a significant difference between baseline and day 4 (MD = −3.10 [95% CI −6.02 to −0.18], *P* = 0.03), baseline and day 21 (MD = −3.30 [95% CI −6.53 to −0.07], *P* = 0.04). There were no significant differences between baseline and day 56 (MD = −3.06 [95% CI −6.64 to 0.53], *P* = 0.14), and no significant differences between day 4 and day 21 (MD = −0.20 [95% CI −2.16 to 1.76], *P* = 1.0), day 4 and day 56 (MD = 0.04 [95% CI −2.64 to 2.72], *P* = 1.0), or day 21and day 56 (MD = 0.24 [95% CI −1.88 to 2.40], *P* = 1.0). There was no significant main effect on the treatment group (*F*_1,96_ = 0.08, *P* = 0.78, MD = 1.04 [95% CI −6.40 to 8.47]) or the living arrangement group (*F*_1,96_ = 0.13, *P* = 0.72, MD = 1.34 [95% CI −6.09 to 8.78]). There was no significant interaction effect between the treatment group and living arrangement group (*F*_1,96_ = 0.0, *P* = 0.99), treatment group and time (*F*_2.2,213_ = 0.45, *P* = 0.66), living arrangement group and time (*F*_2.2,213_ = 0.50, *P* = 0.63), and between time, treatment group, and living arrangement group (*F*_2.2,213_ = 1.63, *P* = 0.20).

**Figure 4.**
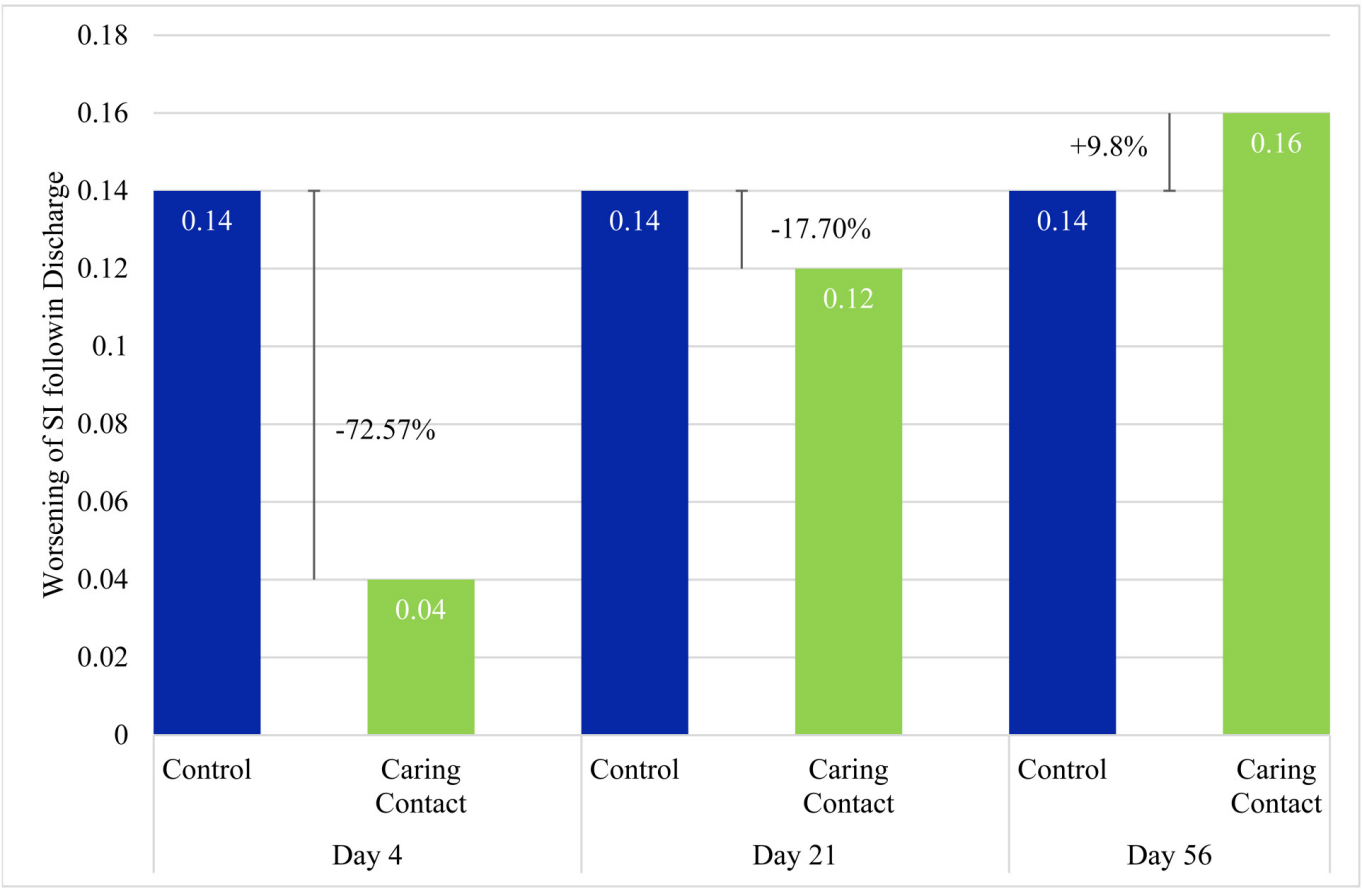
Increase of Suicidal Ideation from Baseline.

The mean Caring Contact group scores from the SI item on the HSCL-25 were 1.3 (*SD* = 0.7) on the baseline, 1.37 (*SD* = 0.7) on day 4, 1.45 (*SD* = 0.7) on day 21, and 1.5 (*SD* = 0.8) on day 56. For the control group, the scores were 1.41 (*SD* = 0.7) on the baseline, 1.6 (*SD* = 0.8) on day 4, 1.6 (*SD* = 0.7) on day 21, and 1.6 (*SD* = 0.8) on day 56. See Figure 4 for group change scores and percent change in SI of the Caring Contact group relative to the control group. The data were badly skewed and not normally distributed so we conducted the related samples Friedman's two-way ANOVA by ranks. There was a trend towards a significant difference in the Caring Contact group (*X*^2^[3] = 7.16, *P* = 0.07) and the control group (*X*^2^[3] = 6.21, *P* = 0.10).

Percentages shown represent the percent change relative to the control group.

We were unable to conduct the same analyses for individuals discharged during mandatory self-isolation versus no mandatory self-isolation since few participants were discharged during no mandatory isolation periods.

## Discussion

This study delivered Caring Contacts to patients following discharge from psychiatric hospitalization during a pandemic. To our knowledge, this is the first study to evaluate the use of Caring Contacts in Canada and one of the only studies to do so during the COVID-19 pandemic.^16^ Notably, the majority of eligible patients consented to participate, which is indicative of feasibility and implied acceptability, especially given the added burden of participation in a research protocol.

Both groups experienced statistically significant worsening of mood and anxiety symptoms post-discharge. This finding is consistent with previous studies that have identified the time period after discharge as a high risk for suicide.^1,2^ Specifically, our findings showed a significant worsening of total symptom severity between baseline and all other time points post-discharge and no significant differences between day 4, day 21, and day 56. This suggests that most of the deterioration observed in the first 2 months post-discharge was observable 4 days post-discharge. This could be the result of a combination of factors including the loss of structure and support of the inpatient unit, and adjusting back to the stress of daily living.^17^ Patients may underestimate their own ability to cope and many may have not yet accessed follow-up resources. During a pandemic, these resources may also be disrupted. Additionally, hospitals often discharge patients while they are still symptomatic, but no longer meet criteria for involuntary admission, and some patients leave against medical advice. These factors may contribute to the most significant deterioration occurring within 4 days. This finding may inform the timing of future interventions given the increase in symptom severity immediately post-discharge.

Our results did not show a statistically significant difference between the Caring Contact group and the control group at any time point (day 4, day 21, and day 56), however, the pilot study was underpowered to show significant differences. The day 4 results identified a 24.2% and 72.6% attenuated worsening in the Caring Contact group for total symptom severity and SI, respectively, compared to the control group. This suggests a possible signal in favour of the Caring Contact group on day 4. Adjustments to the intervention and larger sample size may demonstrate a significant effect. This modest difference between the groups on day 4 suggests that although both groups worsened post-discharge, an intervention like Caring Contacts may help to attenuate this short-term worsening.

The signal in favour of the Caring Contact group faded from a 24.2% difference between groups on day 4 to a 10% difference between groups on day 21 for total symptoms and from 72.6% on day 4 to 17.7% on day 21 for SI. By day 56, the control group had lower change scores and the Caring Contact group had a 5.4% and 9.8% increase relative to the control group in worsening total symptoms and SI, respectively. This could be due to a few reasons. Our intervention may not have been impactful enough in style or content (e.g., no imagery, lack of personalization, email delivery, and frequency of contact), and the content did not change significantly which may have led to disappointment. It is possible that the novelty of the message may have worn off by the time participants received the third communication on day 56. Since the difference between the Caring Contact and the control communication was only in one aspect of the content, receiving a control communication may have provided some benefit compared to not receiving anything post-discharge. Completion rates of the HSCL-25 were about the same between groups at each time point, which suggests that the group participants were assigned did not clearly affect the level of participant engagement in the study. It is also possible that with the between-group difference in marital status we may expect the control group to be more resilient and/or socially supported as the control group had more participants that were married/living common law.^18^

The results of this study should be considered in the context of several limitations. Firstly, given the pilot nature of the study, we are exploring and commenting on findings that are not statistically significant. Therefore, caution is required when interpreting the data. One of the challenges we experienced was a low questionnaire return rate. Reminder emails sent 4 days after the scheduled email and phone calls on day 14 helped to increase return rate, however, at the third communication (day 56) the rate still dropped below 50% for both groups. While we adjusted for this using LOCF, this method is not free from bias. Previous Caring Contacts interventions have used letters,^19^ postcards,^20^ text messages,^7^ phone calls,^21^ and emails.^22^ Using an automated email allowed us to reach a wide group of participants while keeping costs low and improving feasibility. This type of intervention could be easily scaled up and maintained within acute care settings and can potentially work synergistically with other interventions (e.g., enhancing access to care during urgent, high-need periods). However, it is possible that a more direct form of personalized contact, like phone calls, handwritten letters, or individualized text messages, could have improved the return rate and our observed mental health effects may have been more pronounced. Additionally, we were unable to include other confounding factors such as contact with a psychiatrist/family physician/therapist in the primary outcome analysis. Other limitations of our study include that we were not able to see how many participants opened the emails. Similarly, we were not able to measure whether participants used the emergency resources in the email. Furthermore, the only outcome measure was the HSCL-25. Our exploratory SI data was based on a single item from the HSCL-25. While having a single outcome measure improved feasibility, including other scales examining SI and behaviour may have provided more insight and resulted in more robust data.

## Conclusion

In the days following discharge, participants experienced a significant worsening of mental health symptoms. This pilot study did identify a possible signal that receiving a Caring Contact during the early time period post-discharge was associated with an attenuation in worsening of overall symptoms and SI. While there was an insufficient sample size to reach statistically significant differences between groups, these results support the relevance of a further series of studies to investigate the optimal content, format and integration of Caring Contacts for a Canadian clinical population.

## Supplemental Material

sj-docx-1-cpa-10.1177_07067437221121111 - Supplemental material for Caring Contacts to Reduce Psychiatric Morbidity Following Hospitalization During the COVID-19 Pandemic: A Pilot Randomized Controlled Trial Click here for additional data file. Supplemental material, sj-docx-1-cpa-10.1177_07067437221121111 for Caring Contacts to Reduce Psychiatric Morbidity Following Hospitalization During the COVID-19 Pandemic: A Pilot Randomized Controlled Trial by Sarah Holman, Rosalie Steinberg, Mark Sinyor, Hillary Lane, Kaleigh Starritt, Sidney H. Kennedy, Thomas Niederkrotenthaler, Ari Zaretsky, Saulo Castel and Ayal Schaffer in The Canadian Journal of Psychiatry

## References

[1] Chung D Hadzi-Pavlovic D Wang M Swaraj S Olfson M Large M . Meta-analysis of suicide rates in the first week and the first month after psychiatric hospitalisation. BMJ Open. 2019;9(3):e023883. doi:10.1136/bmjopen-2018-023883 PMC6475206 30904843

[2] Chung DT Ryan CJ Hadzi-Pavlovic D Singh SP Stanton C Large MM . Suicide rates after discharge from psychiatric facilities: a systematic review and meta-analysis. JAMA Psychiatry. 2017;74(7):694. doi:10.1001/jamapsychiatry.2017.1044 28564699 PMC5710249

[3] Zalsman G Hawton K Wasserman D , et al. Suicide prevention strategies revisited: 10-year systematic review. Lancet Psychiatry. 2016;3(7):646-659. doi:10.1016/S2215-0366(16)30030-X 27289303

[4] Reger MA Luxton DD Tucker RP , et al. Implementation methods for the caring contacts suicide prevention intervention. Prof Psychol Res Pract. 2017;48(5):369-377. doi:10.1037/pro0000134

[5] Motto JA . Suicide prevention for high-risk persons who refuse treatment. Suicide Life Threat Behav. 1976 Winter;6(4):223-230. 1023455

[6] Luxton DD June JD Comtois KA . Can postdischarge follow-up contacts prevent suicide and suicidal behavior? A review of the evidence. Crisis. 2013;34(1):32-41. doi:10.1027/0227-5910/a000158 22846445

[7] Comtois KA Kerbrat AH Decou CR , et al. Effect of augmenting standard care for military personnel with brief caring text messages for suicide prevention: a randomized clinical trial. JAMA Psychiatry. 2019;76(5):474. doi:10.1001/jamapsychiatry.2018.4530 30758491 PMC6495345

[8] Pfefferbaum B North CS . Mental health and the COVID-19 pandemic. N Engl J Med. 2020;383(6):510-512. doi:10.1056/nejmp2008017 32283003

[9] Wind TR Rijkeboer M Andersson G Riper H . The COVID-19 pandemic: the ‘black swan’ for mental health care and a turning point for e-health. Internet Interv. 2020;20:100317. doi:10.1016/j.invent.2020.100317 32289019 PMC7104190

[10] Patten SB Kutcher S Streiner D Gratzer D Kurdyak P Yatham L . Population mental health and COVID-19: why do we know so little? Can J Psychiatry. 2021;66(9):782-784. doi:10.1177/07067437211010523 33871302 PMC8495301

[11] Kim HK Carvalho AF Gratzer D , et al. The impact of COVID-19 on psychiatric emergency and inpatient services in the first month of the pandemic in a large urban mental health hospital in Ontario, Canada. Front Psychiatry. 2021;12. doi:10.3389/fpsyt.2021.563906 PMC8102788 33967842

[12] Hao F Tan W Jiang L , et al. Do psychiatric patients experience more psychiatric symptoms during COVID-19 pandemic and lockdown? A case-control study with service and research implications for immunopsychiatry. Brain Behav Immun. 2020;87:100-106. doi:10.1016/j.bbi.2020.04.069 32353518 PMC7184991

[13] Harris PA Taylor R Thielke R Payne J Gonzalez N Conde JG . Research electronic data capture (REDCap)—a metadata-driven methodology and workflow process for providing translational research informatics support. J Biomed Inform. 2009;42(2):377-381. doi:10.1016/j.jbi.2008.08.010 18929686 PMC2700030

[14] Harris PA Taylor R Minor BL , et al. The REDCap consortium: building an international community of software platform partners. J Biomed Inf. 2019;95:103208. doi:10.1016/j.jbi.2019.103208 PMC7254481 31078660

[15] Hesbacher PT Rickels K Morris RJ Newman H Rosenfeld H . Psychiatric illness in family practice. J Clin Psychiatry. 1980 Jan;41(1):6-10. 7351399

[16] Landes SJ Jegley SM Kirchner JAE , et al. Adapting caring contacts for veterans in a department of veterans affairs emergency department: results from a type 2 hybrid effectiveness-implementation pilot study. Front Psychiatry. 2021;12. doi:10.3389/fpsyt.2021.746805 PMC8548725 34721114

[17] Owen-Smith A Bennewith O Donovan J , et al. “When you’re in the hospital, you’re in a sort of bubble.” Understanding the high risk of self-harm and suicide following psychiatric discharge: a qualitative study. Crisis. 2014;35(3):154-160. doi:10.1027/0227-5910/a000246 24698726

[18] Agerbo E Qin P Mortensen PB . Psychiatric illness, socioeconomic status, and marital status in people committing suicide: a matched case-sibling-control study. J Epidemiol Community Health. 2006;60(9):776-781. doi:10.1136/jech.2005.042903 16905722 PMC2566026

[19] Motto JA Bostrom AG . A randomized controlled trial of postcrisis suicide prevention. Psychiatry Serv. 2001;52(6):828-833. doi:10.1176/appi.ps.52.6.828 11376235

[20] Carter GL Clover K Whyte IM Dawson AH D’Este C . Postcards from the EDge project: randomised controlled trial of an intervention using postcards to reduce repetition of hospital treated deliberate self poisoning. Br Med J. 2005;331(7520):805. doi:10.1136/bmj.38579.455266.E0 16183654 PMC1246077

[21] Vaiva G Ducrocq F Meyer P , et al. Effect of telephone contact on further suicide attempts in patients discharged from an emergency department: randomised controlled study. Br Med J. 2006;332(7552):1241-1245. doi:10.1136/bmj.332.7552.1241 16735333 PMC1471935

[22] Luxton DD Smolenski DJ Reger MA Relova RM V Skopp NA . Caring E-mails for military and veteran suicide prevention: a randomized controlled trial. Suicide Life Threat Behav. 2020;50(1):300-314. doi:10.1111/sltb.12589 31562660

