# Supplementary material for: Caring Contacts to Reduce Psychiatric Morbidity Following Hospitalization During the COVID-19 Pandemic: A Pilot Randomized Controlled Trial
Source: Can J Psychiatry. 2022 Aug 22;68(3):152–62. doi: 10.1177/07067437221121111 (PMC9412148; doi:10.1177/07067437221121111)
Supplement: sj-docx-1-cpa-10.1177_07067437221121111 - Supplemental material for Caring Contacts to Reduce Psychiatric Morbidity Following Hospitalization During the COVID-19 Pandemic: A Pilot Randomized Controlled Trial [file sj-docx-1-cpa-10.1177_07067437221121111.docx]

**Caring Contact Email 1**

Dear ____,

Thank you for agreeing to participate in our Caring Contacts Research Study. This is our first follow up email to you.

You have recently left our care at Sunnybrook. We are invested in your health and wellness and wish you continued strength in your health care journey. You might feel alone out there, however there are opportunities to find encouraging words in recovery. Be on the lookout for them, search them out and really let them sink in.

We would like to know how you are doing now. We ask that you please take a moment to fill out this brief questionnaire. You may open the questionnaire in your web browser by clicking the link below:
Symptom Questions

If you are feeling distressed or isolated there is help available in the community. If needed, you can call the Gerstein Centre at 416-929-5200, or the National Distress Line at 1-833-456-4566 (or text 45645 or visit <https://www.crisisservicescanada.ca/en/call-us/>).

Take good care,
From your care team at Sunnybrook Health Sciences Centre


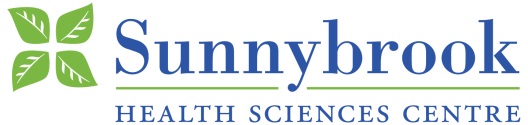


If the link above does not work, try copying the link below into your web browser:
<https://redcap.sunnybrook.ca/redcap/surveys/?s=7SqPgdpqTv>

**Caring Contact Email 2**

Dear ____,

Thank you for agreeing to participate in our Caring Contacts Research Study. This is our second follow up email to you.

This email is to let you know we wanted to reach out again following your discharge from hospital. Your team at Sunnybrook is sending you good wishes in your continued recovery. It is worthwhile to remember your emotional health takes time and you gain more wisdom with each new experience. Remember the most encouraging words you’ll hear are those you are able to tell yourself.

We would like to know how you are doing now. We ask that you please take a moment to fill out this brief questionnaire. You may open the questionnaire in your web browser by clicking the link below: Symptom Questions

If you are feeling distressed or isolated there is help available in the community. If needed, you can call the Gerstein Centre at 416-929-5200, or the National Distress Line at 1-833-456-4566 (or text 45645 or visit <https://www.crisisservicescanada.ca/en/call-us/>).

Take good care,
From your care team at Sunnybrook Health Sciences Centre


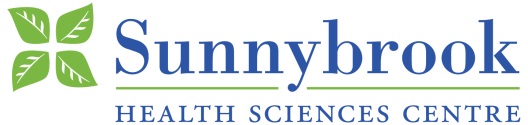


If the link above does not work, try copying the link below into your web browser: <https://redcap.sunnybrook.ca/redcap/surveys/?s=7SqPgdpqTv>

**Caring Contact Email 3**

Dear ____,

Thank you for agreeing to participate in our Caring Contacts Research Study. This is our third follow up email to you.

Hello, this is your health care team at Sunnybrook reaching out to you again. We sincerely hope you are able to use your skills to take care of yourself because you are a truly valuable person. The more you learn, the more you grow. The more you grow, the stronger your recovery foundation becomes. We wanted to let you know this will be our last communication with you as part of this series but do remember we wish you well in your continued recovery.

We would like to know how you are doing now. We ask that you please take a moment to fill out this brief questionnaire. You may open the questionnaire in your web browser by clicking the link below: Symptom Questions

If you are feeling distressed or isolated there is help available in the community. If needed, you can call the Gerstein Centre at 416-929-5200, or the National Distress Line at 1-833-456-4566 (or text 45645 or visit <https://www.crisisservicescanada.ca/en/call-us/>).

Take good care,
From your care team at Sunnybrook Health Sciences Centre


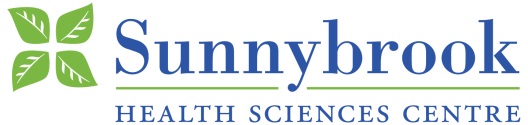


If the link above does not work, try copying the link below into your web browser: <https://redcap.sunnybrook.ca/redcap/surveys/?s=7SqPgdpqTv>

**Control Group Email 1**

Dear ____,

Thank you for agreeing to participate in our Caring Contacts Research Study. This is our first follow up email to you.

We would like to know how you are doing now. We ask that you please take a moment to fill out this brief questionnaire. You may open the questionnaire in your web browser by clicking the link below:
Symptom Questions

If you are feeling distressed or isolated there is help available in the community. If needed, you can call the Gerstein Centre at 416-929-5200, or the National Distress Line at 1-833-456-4566 (or text 45645 or visit <https://www.crisisservicescanada.ca/en/call-us/>).

Take good care,
From your care team at Sunnybrook Health Sciences Centre


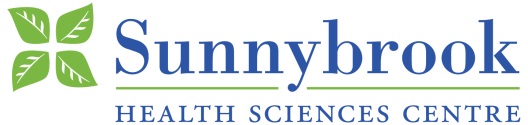


If the link above does not work, try copying the link below into your web browser:
<https://redcap.sunnybrook.ca/redcap/surveys/?s=7SqPgdpqTv>

**Control Group Email 2**

Dear ____,

Thank you for agreeing to participate in our Caring Contacts Research Study. This is our second follow up email to you.

We would like to know how you are doing now. We ask that you please take a moment to fill out this brief questionnaire. You may open the questionnaire in your web browser by clicking the link below:
Symptom Questions

If you are feeling distressed or isolated there is help available in the community. If needed, you can call the Gerstein Centre at 416-929-5200, or the National Distress Line at 1-833-456-4566 (or text 45645 or visit <https://www.crisisservicescanada.ca/en/call-us/>).

Take good care,
From your care team at Sunnybrook Health Sciences Centre


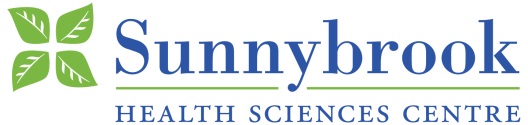


If the link above does not work, try copying the link below into your web browser:
<https://redcap.sunnybrook.ca/redcap/surveys/?s=7SqPgdpqTv>

**Control Group Email 3**

Dear ____,

Thank you for agreeing to participate in our Caring Contacts Research Study. This is our third follow up email to you.

We would like to know how you are doing now. We ask that you please take a moment to fill out this brief questionnaire. You may open the questionnaire in your web browser by clicking the link below:
Symptom Questions

If you are feeling distressed or isolated there is help available in the community. If needed, you can call the Gerstein Centre at 416-929-5200, or the National Distress Line at 1-833-456-4566 (or text 45645 or visit <https://www.crisisservicescanada.ca/en/call-us/>).

Take good care,
From your care team at Sunnybrook Health Sciences Centre


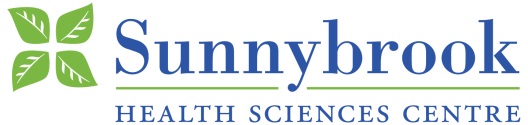


If the link above does not work, try copying the link below into your web browser:
<https://redcap.sunnybrook.ca/redcap/surveys/?s=7SqPgdpqTv>
